# A dataset on concurrent and immediate retrospective measures of sensory perception and preferences of dark chocolates

**DOI:** 10.1016/j.dib.2023.109314

**Published:** 2023-06-14

**Authors:** Michel Visalli, Benjamin Mahieu, Pascal Schlich

**Affiliations:** a Centre des Sciences du Goût et de l'Alimentation, Institut Agro Dijon, CNRS, INRAE, Université Bourgogne, F-21000 Dijon, France; b INRAE, PROBE research infrastructure, ChemoSens facility, F-21000 Dijon, France; c StatSC, ONIRIS, INRAE, Nantes, France

**Keywords:** Temporal dominance of sensations, Attack-evolution-finish, Ecological measures, Cognitive biases, Sensometrics

## Abstract

This article describes data related to the research paper entitled “Concurrent vs. retrospective temporal data collection: Attack-evolution-finish as a simplification of Temporal Dominance of Sensations?” [1]. Temporal sensory perception data of five dark chocolates that vary in cocoa content were collected from 129 consumers who evaluated the samples in two sessions, using a different sensory evaluation method in each session. A within-subject design was set-up to compare the two data collection methods: consumers in Panel 1 (36 men and 32 women aged 19 to 63 years old) started with the Temporal Dominance of Sensations (TDS) method, and consumers in Panel 2 (35 men and 26 women aged 19 to 61 years old) started with the Attack-Evolution-Finish dominance (AEF-D) method. For each chocolate, consumers had to report the sensations they perceived either concurrently (TDS) or retrospectively (AEF-D) to the tasting. After the descriptive task, consumers were asked to rate their liking for chocolates on a 9-point discrete scale. Finally, consumers had to answer questions related to the difficulty of the descriptive task. The dataset includes information on consumers’ gender, age and frequency of consumption of dark chocolates. The dataset can be reused by sensometricians to compare methods or develop new statistical models for data analysis. It can also be reused to compare at the individual level declarative sensory measures collected either concurrently or retrospectively to tasting. Thus, the impact of cognition (due to memorization, stress or complexity of measurements) on sensory description and liking can be investigated. More specifically, this dataset can be help understand how the dynamics of perception of texture, mouthfeel and flavour attributes are integrated when using static measures.

**Specifications Table**

**Table:** 

Subject	Food science
Specific subject area	Temporal sensory evaluation
Type of data	Table Figure
How the data were acquired	Two consumer panels (129 consumers in total) evaluated the samples in the sensory booths of the ChemoSens platform, using the TimeSens© [3] software, version 2.0. Two temporal sensory evaluation methods were used by consumers to describe their temporal perception: Temporal Dominance of Sensation (TDS [4]) and Attack-Evolution-Finish Dominance (AEF-D [1]). Liking scores were rated using 11-point discrete scales (between 0 and 10). Items related to the perception of the tasks were evaluated using 5-point Likert scales [5].
Data format	Raw
Description of data collection	In two sessions spaced 48 hours apart, five samples of dark chocolates were evaluated by all consumers separated in two panels (same samples in each session, within-subject design). The consumers had first to qualitatively describe their temporal perception of each sample by selecting in a predefined list of attributes (Astringent, Bitter, Cocoa, Dry, Fat, Floral, Fruity, Melting, Sour, Sticky, Sweet, Woody) those they perceived as dominant. Half started with TDS in session 1, half by AEF-D, and the order was reversed in session 2. When using TDS, the evaluation task was concurrent to the tasting, the times of citations of each attribute and the duration of the tasting were recorded. When using AEF-D, the evaluation task was retrospective to the tasting and only the duration of tasting was recorded. After the descriptive task, without re-tasting the samples, the consumers had to rate their liking for the sample, and the time it took was recorded. After having evaluated the five samples, the consumers filled a questionnaire about the difficulty of the descriptive task. At the end of session 2, they reported their opinion on the relative difficulty of each descriptive task.
Data source location	Institution: INRAE City/Town/Region: Dijon Country: France
Data accessibility	Repository name: Mendeley data Data identification number: 10.17632/9c9g3rh8rd.1[2] Direct URL to data: https://data.mendeley.com/datasets/9c9g3rh8rd/1
Related research article	M. Visalli, B. Mahieu, A. Thomas, P. Schlich, Concurrent vs. retrospective temporal data collection: Attack-evolution-finish as a simplification of Temporal Dominance of Sensations? Food Quality and Preference, 85, (2020), 103956, ISSN 0950-3293. https://doi.org/10.1016/j.foodqual.2020.103956.

## Value of the Data

• These data are useful because they allow a comparison at the panel and individual levels of concurrent and retrospective descriptive and hedonic measures collected with sensory evaluation methods.
• The food science and sensometrics community can benefit from these data to gain insight on sensory perception of dark chocolates or test new statistical models, respectively.
• Researchers in cognitive science can also benefit from these data for testing if Kahneman's theory [6] (fast vs. slow thinking) applies on sensory perception.
• These data can be reused to investigate individual differences in perception due to cognitive biases (complexity of the instructions, memorization, stress) and related to retrospective and concurrent measures, and test if all subjects are affected by these biases equally or differently (depending on individual characteristics).
• They can also be reused for studying differences between what is perceived (concurrent measures) and what is recalled and integrated (retrospective measures) according to sensory modalities (differences between texture, mouthfeel, basic taste and aroma attributes), and how these differences affect hedonic perception.

## 1 Objective

As sensory perception is a dynamic process, sensory evaluation methods such as TDS have been developed to collect data simultaneously with tasting. However, if the dynamic evaluation task is natural, it remains demanding for consumers as it attaches great importance to the moment at which the sensations are cited. To ensure valid measures and limit heterogeneity in consumers’ behaviours with TDS, a familiarization step with the method may be required, which is not always possible outside of laboratory settings. The published article introduced AEF-D as an alternative, simplified method for temporal measurements. It focused on methods comparison, practical aspects of data collection and statistical analysis related to discrimination of products at the panel level. However, the data collected can also be used for addressing questions related to the subjects and the descriptors. More precisely, it is possible to study if individual differences can be observed about retrospective integration of dynamic perception, and if this integration varies according to the sensory modalities and affects the liking. These questions not covered in the published research article are of primary importance because they are linked to the ecological validity of the measures.

## 2 Data Description

The dataset is provided as an Excel file (data.xlsx) including seven sheets.

***Subjects*** provides information about the consumers.

“Panel”: name of the panel to which the participant has been assigned.

“Subject”: unique code of the participant.

“Gender”: gender of the participant (M: male or F: female).

“Age”: age of the participant.

***Products*** provides information about the dark chocolates evaluated by the participants.

“Product”: code of the product. The 2-digit number corresponds to the percentage of cocoa.

***TDS*** and ***AEF-D*** provide data collected with the corresponding temporal evaluation methods.

“Subject”, “Product”: see above.

“Attribute”: dominant attribute clicked by the participant (Astringent, Bitter, Cocoa, Dry, Fat, Floral, Fruity, Melting, Sour, Sticky, Sweet, Woody + START and STOP in TDS).

“Time”: time (in seconds) of click on the attribute (TDS)

“Period”: period (A for Attack, E for Evolution, F for finish) during which the attribute was retrospectively declared applicable (AEF-D).

***AEF-D tasting duration*** provide data about the duration of the perception before the AEF-D evaluation.

“Subject”, “Product”: see above.

“Duration”: duration of perception (in seconds).

***Liking*** provide data about the liking scores given after the descriptive task.

“Subject”, “Product”: see above.

“AfterTDS-Liking” and “AfterAEF-Liking”: value rated on a 11-point discrete scale (between 0 and 10) after the TDS and AEF task, respectively.

“AfterTDS-TimeToReportLiking” and “AfterAEF-TimeToReportLiking”: time (in seconds) required to report the liking score.

***Questionnaire*** provide answers of the participants to task-related questions. Items measured on a Likert scale used the following labels: “strongly agree” (5), “agree” (4), “neither agree nor disagree” (3), “disagree” (2), and “strongly disagree” (1).

“Subject”: see above.

“ExperienceWithTDS”: previous experience of the participant with the TDS method (Yes, No or Don't know)

“AfterTDS-Q1”: Answer to the question “the oral explanations were useful” (Likert scale, TDS only).

“AfterTDS-Q2”: Answer to the question “The explanations displayed on the screen about how to evaluate chocolates were useful” (Likert scale, TDS only).

“AfterTDS-Q3” and “AfterAEF-Q3”: Answers to the question “I understood how to evaluate the chocolates” (Likert scale).

“AfterTDS-Q4” and “AfterAEF-Q4”: Answers to the question “The list of sensations was exhaustive” (Likert scale).

“AfterTDS-Q5” and “AfterAEF-Q5”: Answers to the question “The sensations were sufficiently explanatory” (Likert scale).

“AfterTDS-Q7A”: Answer to the question “It was easy to identify the sensations that caught my attention during the tasting” (Likert scale, TDS only).

“AfterTDS-Q8”: Answer to the question “It was easy to quickly click on a sensation when it caught my attention” (Likert scale, TDS only).

“AfterTDS-Q9”: Answer to the question “It was easy to identify when to click STOP” (Likert scale, TDS only).

“AfterTDS-Q10” and “AfterAEF-Q10”: Answers to the question “The order in which I listed the sensations was important” (Likert scale).

“AfterTDS-Q11” and “AfterAEF-Q11”: Answers to the questions “I could list the same feeling several times” (Likert scale).

“AfterTDS-Q12” and “AfterAEF-Q12”: Answers to the questions “The questionnaire interface was easy to use” (Likert scale).

“AfterTDS-Q13” and “AfterAEF-Q13”: Answers to the questions “The task that was asked of me was easy” (Likert scale).

“AfterAEF-Q1”: Answer to the question “I wish I had oral explanations” (Likert scale, AEF-D only).

“AfterAEF-Q2”: Answer to the question “The explanations displayed on the screen about how to evaluate chocolates were sufficient” (Likert scale, AEF-D only).

“AfterAEF-Q6”: Answer to the question “I wished I could select more than 3 sensations” (Likert scale, AEF-D only).

“AfterAEF-Q7B”: Answer to the question “It was easy to identify the sensations perceived at the beginning of the tasting” (Likert scale, AEF-D only).

“AfterAEF-Q7C”: Answer to the question “It was easy to identify the sensations perceived at the middle of the tasting” (Likert scale, AEF-D only).

“AfterAEF-Q7D”: Answer to the question “It was easy to identify the sensations perceived at the end of the tasting” (Likert scale, AEF-D only).

“RelativeDifficulty”: Answer to the question “Compared to the task in the first session, did today's task seem to you to be ‘much easier’, ‘easier’, ‘at the same level of difficulty’, ‘more difficult’, or ‘much more difficult?’”. Coded between -2 (TDS much easier) and 2 (AEF much easier).

“Comment”: open-ended question about the overall opinion of the participant about the experiment (in French).

**Fig. 1:**
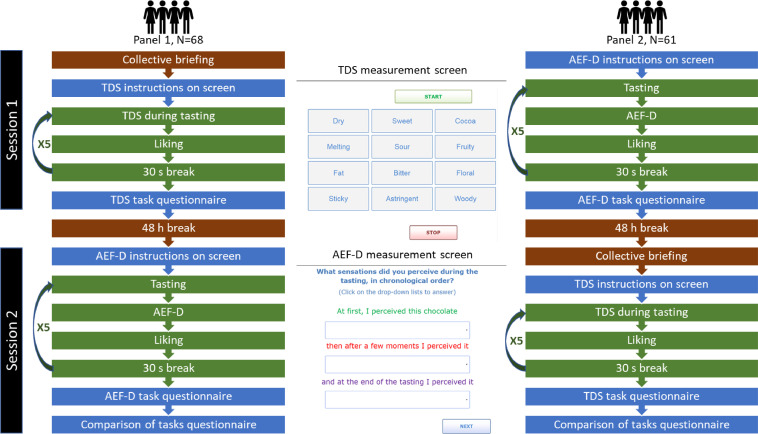
Procedure of data collection.

Fig. 1 describes the procedure of data collection.

## 3 Experimental Design, Materials and Methods

### 3.1 Participants

Participants were preselected from a population registered in the ChemoSens Platform's PanelSens database (declared to the relevant authority, Commission Nationale Informatique et Libertés – CNIL, authorization number 1148039). The inclusion conditions for participating in this study were as follows: being between 18 and 65 years old; not suffering from food or non-food allergies; not being pregnant or breastfeeding and not following a restrictive diet incompatible with the consumption of sugar; being a regular consumer of dark chocolate (at least once every two weeks). The purpose of the study was explained via an information sheet sent by email. The participants have to accept the conditions and fill out a written informed consent form before to be included.

One hundred and forty consumers were selected and randomly assigned to one of two panels, with a constraint of balance in gender and age between panels. Due to attrition, a total of 129 consumers (71 men and 58 women, between 19 and 63 years old) finally participated in this study (68 in panel 1, 61 in panel 2). They were compensated for their participation in the study (vouchers worth 20€).

### 3.2 Samples

Five dark chocolates (provided by Barry Callebaut, cocoa product manufacturer), varying in cocoa content (54%, 65%, 68%, 70% and 73%) and in origin of cocoa bean, were selected for this study. The samples were given to the consumers in transparent plastic containers containing 4 callets of chocolates of 0.5 g each and labelled with 3-digit codes.

### 3.3 Data Collection

The consumers participated in sessions of approximately one hour in the sensory lab of ChemoSens at the Centre for Taste and Feeding Behaviour, Dijon, France.

In session 1, consumers in panel 1 firsts attended a collective briefing (groups of 16 persons) aiming at explaining the procedure and demonstrating how to report their perception using the TDS method. The concept of dominance was presented as “the sensation that catches the attention at any time”, and the panel leader shown how to interact with a TDS measurement screen while simulating a tasting. To ensure their understanding of the task, the participants were invited to ask any question.

Then, they were installed in individual booths equipped with computers running TimeSens© V2 software, the data acquisition program. The instructions were reminded on the screen: “You will describe each chocolate by clicking at any moment on the sensation that catches your attention. A sensation can be clicked several times or never. There are no constraints on the number of sensations clicked. You will have to click on START at the same time you put the chocolate in your mouth and on STOP when you no longer perceive anything”.

The sequence presented in green on Fig. 1 was repeated for each chocolate, the samples being presented under white light, at ambient temperature, in a sequential monadic order according to a Williams Latin square. First, the sensory attribute list (chosen based on previous experiments) was displayed on the screen. This list included the following attributes: astringent, bitter, cocoa, dry, fat, floral, fruity, melting, sour, sticky, sweet, woody. The consumers were reminded to familiarize themselves with the location of the attributes on the screen before clicking on the button START (which triggered the chronometer) while at the same time consuming the four callets in a single intake. Once they have clicked on the button STOP (which stopped the chronometer), the consumers had to report their liking on a 11-point discrete scale labeled from 0 (“I did not like at all”) to 10 (“I liked it very much”). Then, a 30-s pause was imposed, during which consumers were asked to rinse their mouths with mineral water.

After having evaluated the five samples, the consumers had to fill a questionnaire related to the TDS evaluation task. The questionnaire included 12 items (see Section “data description>questionnaire”) to evaluate using a 5-point Likert (labels: “strongly agree”, “agree”, “neither agree nor disagree”, “disagree”, and “strongly disagree”). That ended the session, and consumers were invited to come back 48 hours later, at the same time, for session 2.

In session 2, consumers in panel 1 were directly installed in individual booths. The instructions for the AEF-D task were presented on the first screen of the software: “You are going to taste 5 chocolates. After each tasting, we will ask you to choose from a list the 3 sensations that you perceived during the tasting, in the chronological order in which you perceived them. Here is the list of sensations available: astringent, bitter, cocoa, dry, fat, floral, fruity, melting, sour, sticky, sweet, woody”. The second screen presented examples of description: “Example: At first, I perceived this chocolate sour, then after a few moments I perceived it fruity, and at the end of the tasting I perceived it sweet. You can use the same sensation several times; for example: At first, I perceived this chocolate sour, then after a few moments I perceived it sour, and at the end of the tasting I perceived it sweet”.

The sequence presented in green on Fig. 1 was repeated for each chocolate (with the same experimental design as TDS). The consumers were instructed to consume in a single intake the four callets while clicking on the START button at the same time. Then, they were invited to focus and memorize the sensations they perceived. When they did not perceive any sensation, they had to click on the STOP button (which was enabled after 10 s). It was only then that the AEF-D measurement screen allowing them to report the perceived sensations appeared. The instructions were: “What sensations did you perceive during the tasting, in chronological order? (Click on the drop-down lists to answer). At first, I perceived this chocolate…, then after a few moments I perceived it…, and at the end of the tasting I perceived it…”. After the descriptive task, the consumers had to report their liking (same as with TDS) and a 30-s pause was imposed to rinse their mouths.

After having evaluated the five samples, the consumers had to fill a questionnaire related to the AEF-D evaluation task. The questionnaire included 13 items (see Section “data description>questionnaire”). Finally, a last question invited the consumers to compare the relative difficulty of TDS and AEF-D: “Compared to the task in the first session, did today's task seem to you to be ‘much easier’, ‘easier’, ‘at the same level of difficulty’, ‘more difficult’, or ‘much more difficult?’”. They also had the possibility to report anything related to the task using an open-ended question.

For consumers in panel 2, the order of tasks was reversed: AEF-D in session 1, and TDS in session 2.

### Ethics Statements

Each participant was informed of the conditions for participating and validated a consent form. The research was carried out in conformity with the Declaration of Helsinki.

## CRediT authorship contribution statement

**Michel Visalli:** Data curation, Writing – original draft, Visualization. **Benjamin Mahieu:** Investigation, Writing – review & editing. **Pascal Schlich:** Writing – review & editing, Supervision.

## Declaration of Competing Interest

The authors declare that they have no known competing financial interests or personal relationships that could have appeared to influence the work reported in this paper.
